# Supplementary material for: Microbially mediated mechanisms underlie soil carbon accrual by conservation agriculture under decade-long warming
Source: Nat Commun. 2024 Jan 8;15:377. doi: 10.1038/s41467-023-44647-4 (PMC10774409; doi:10.1038/s41467-023-44647-4)
Supplement: Supplementary file 1 — Supplementary Information [file 41467_2023_44647_MOESM1_ESM.pdf]

Supplementary information for

Microbially mediated mechanisms underlie soil carbon accrual by conservation agriculture under decade-long warming

Jing Tian, Jennifer A. J. Dungait, Ruixing Hou, Ye Deng, Iain P. Hartley, Yunfeng Yang, Yakov Kuzyakov, Fusuo Zhang, M. Francesca Cotrufo, Jizhong Zhou

This file includes:
Supplementary Figures 1-10
Supplementary Tables 1 to 10

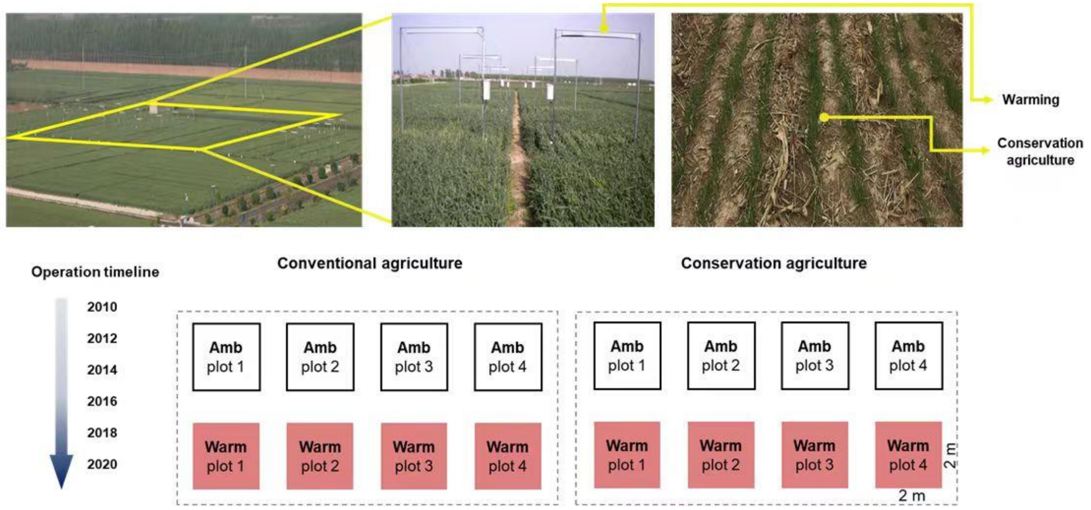

Supplementary Figure 1 Field experimental design. The field experiment had four treatments including conventional agriculture without warming (Conven-Amb), conventional agriculture with warming (Conven-Warm), conservation agriculture without warming (Conserv-Amb) and conservation agriculture with warming (Conserv-Warm).

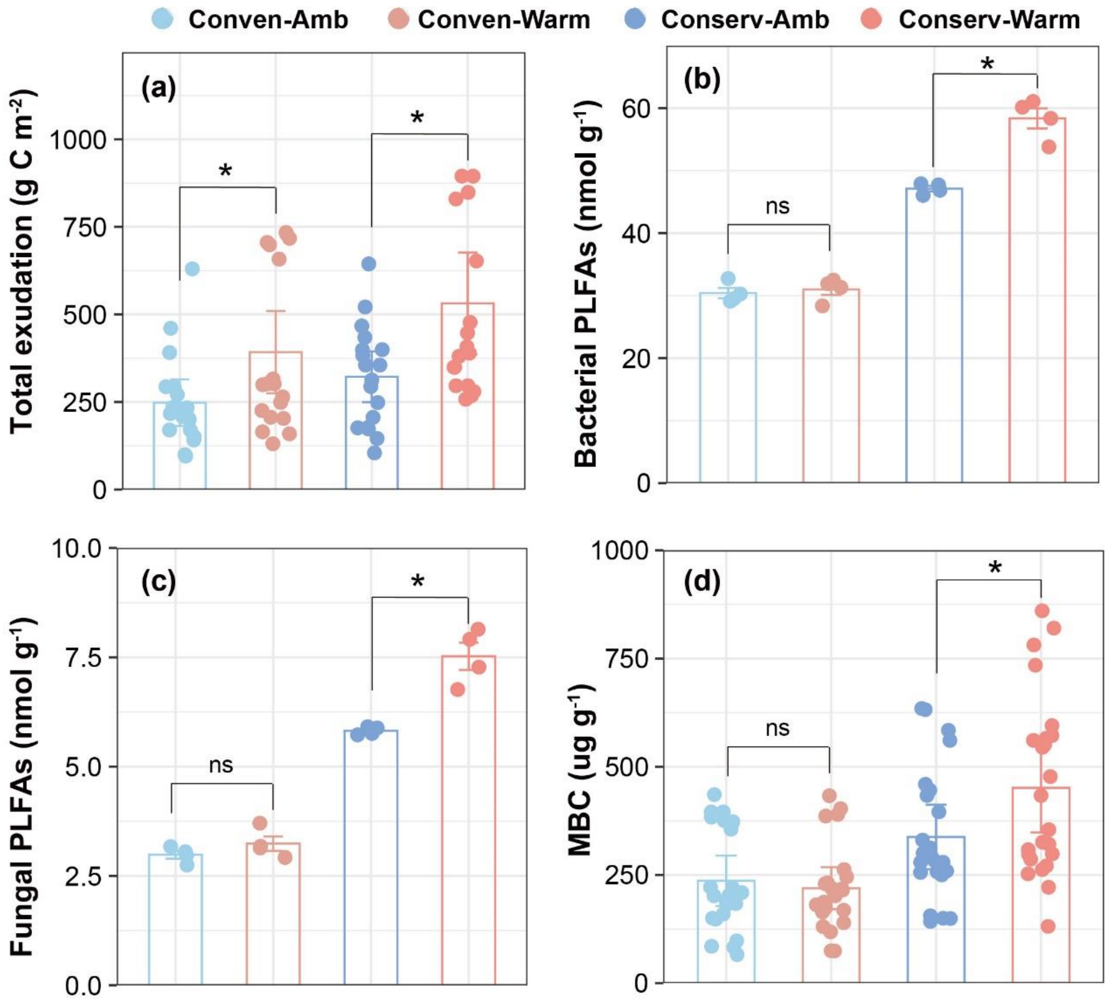

Supplementary Figure 2 The root exudation C input (a), bacterial (b), fungal (c) and total microbial biomass (d) depending on soil warming and management systems. Bars indicate mean ± s.e.m. (n = 15, 4, 4, and 24 independent soil samples per treatment for total exudation, bacterial, fungal, and total microbial biomass, respectively). Two-sided , statistical tests were used to evaluate the data. Asterisks indicate significant differences in the warming effect of individual management as compared with their matched no-warming condition.

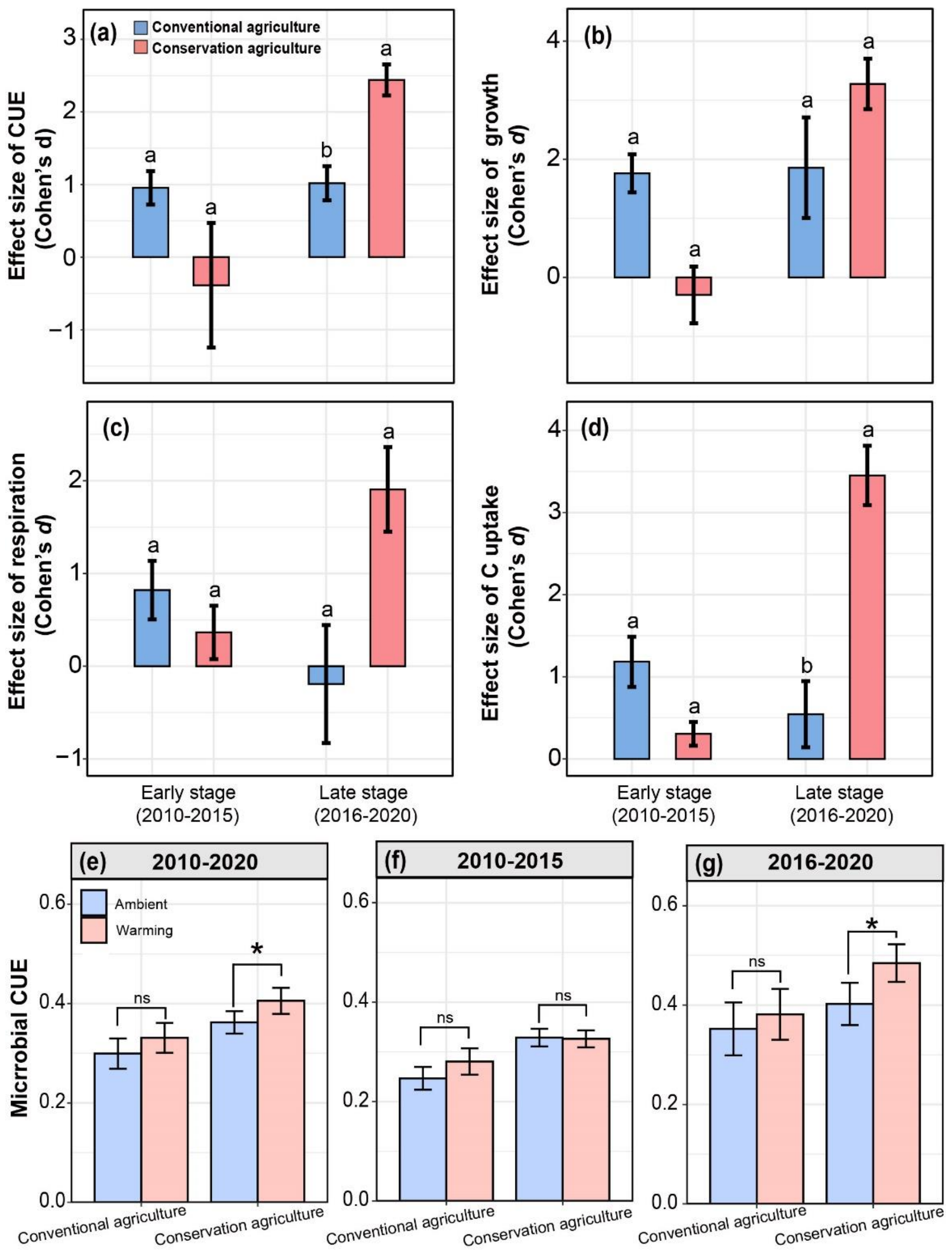

Supplementary Figure 3 Effect size of warming on microbial CUE, growth, respiration and C uptake during early and late stages in conservation and conventional agriculture (a-d). Bars indicate mean ± s.e.m. (n = 12 paired soil samples per management per stage). **Average (2010-2020) and five-year rolling mean (2010-2015; 2016-2020) of CUE depending on warming and management systems (e-g).** Bars indicate mean ± s.e.m. (n = 12 independent soil samples per treatment per stage). Two-sided statistical tests were used to evaluate the data. The different letters for the same stage of the experiment represent significant differences between the two systems at $p < 0.05$. Asterisks indicate significant differences in the warming effect of individual management system as compared with the corresponding no-warming condition.

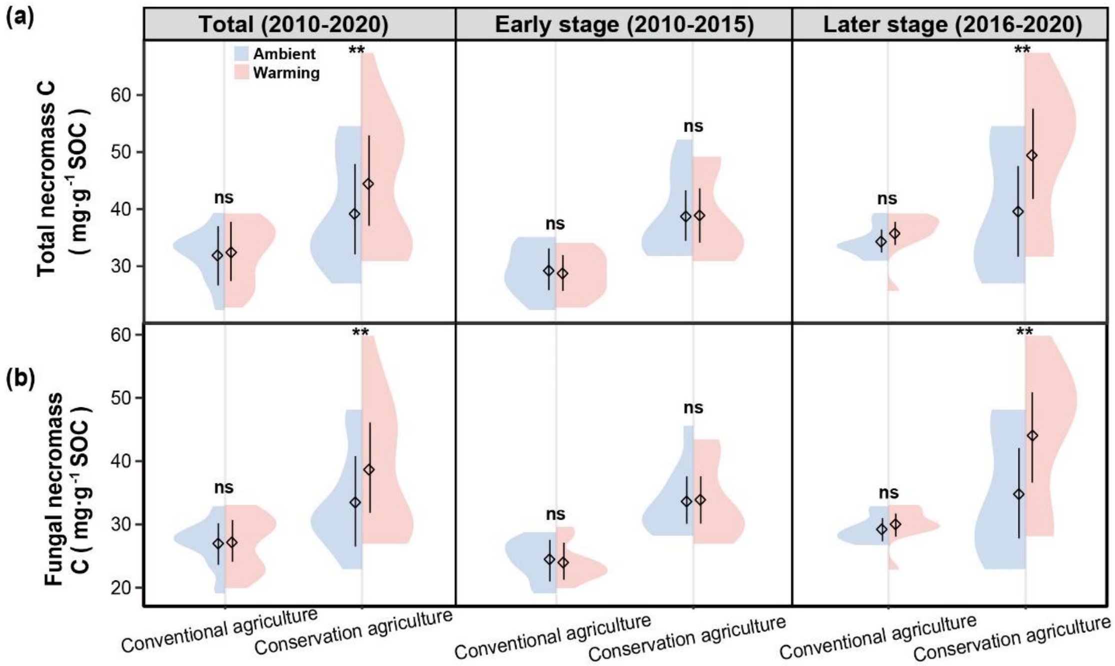

Supplementary Figure 4 Contribution of microbial necromass to SOC in different stages depending on soil warming and management systems. Data are presented as violin plots with mean values ± s.e.m (n = 12 paired soil samples per management per stage). Two-sided statistical tests were used to evaluate the data. Asterisks indicate significant differences in the warming effect of individual management system as compared with the corresponding no-warming condition.

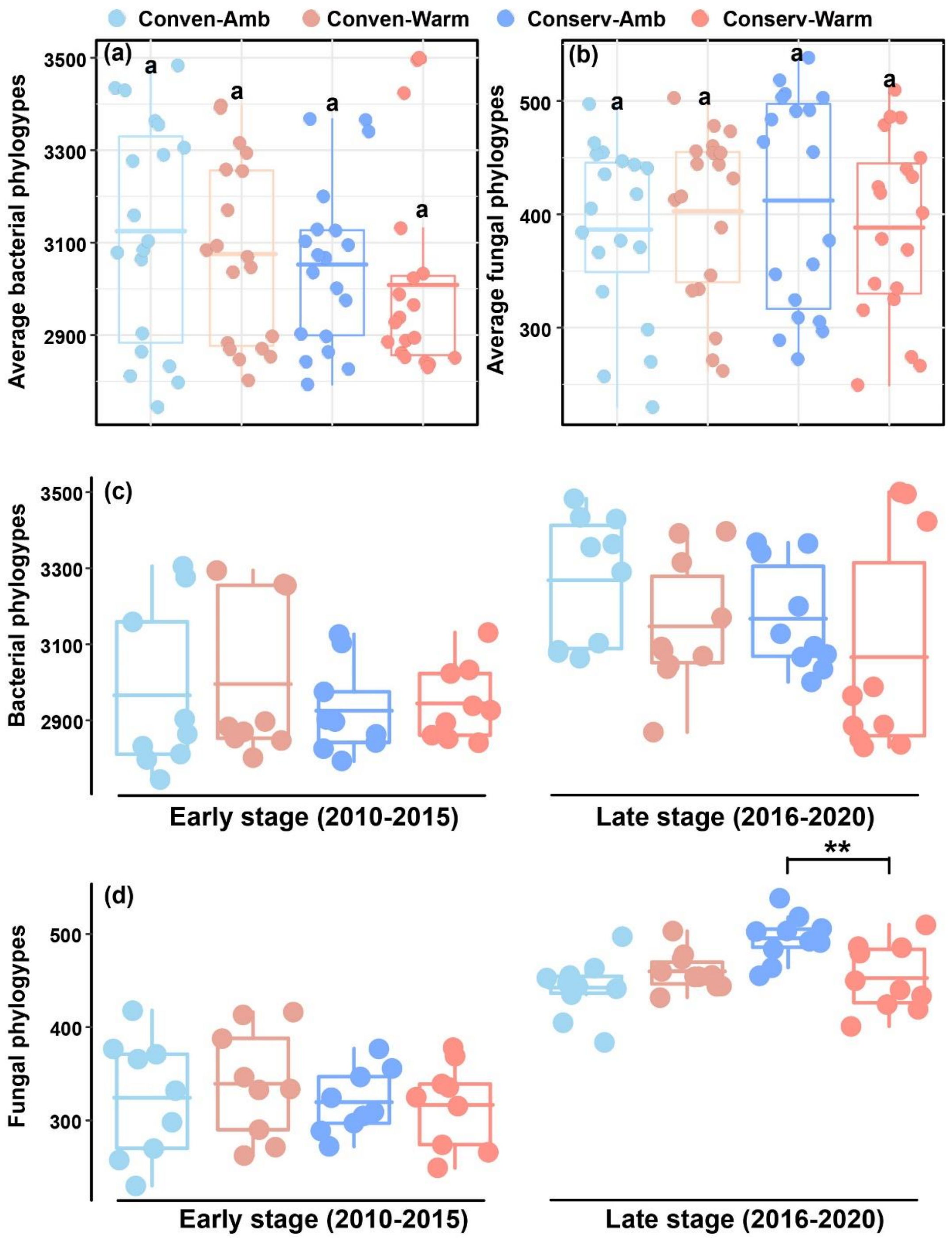

Supplementary Figure 5 Average (a, b) and five-year rolling mean (c, d) of soil bacterial and fungal phylotypes depending on soil warming and management system. Two-sided statistical tests were used to evaluate the data (n = 9 paired soil samples per management per stage). Different letters indicate significant differences among the four treatments. Asterisks indicate significant differences in the warming effect of individual tillage system as compared with the corresponding no-warming condition. The boxplots depict median, first and third quartiles, and full ranges (bounded at 1.5 × interquartile range). Conven-Amb: conventional agriculture without warming; Conven-Warm: conventional agriculture with warming; Conserv-Amb: conservation agriculture without warming; Conserv-Warm: conservation agriculture with warming.

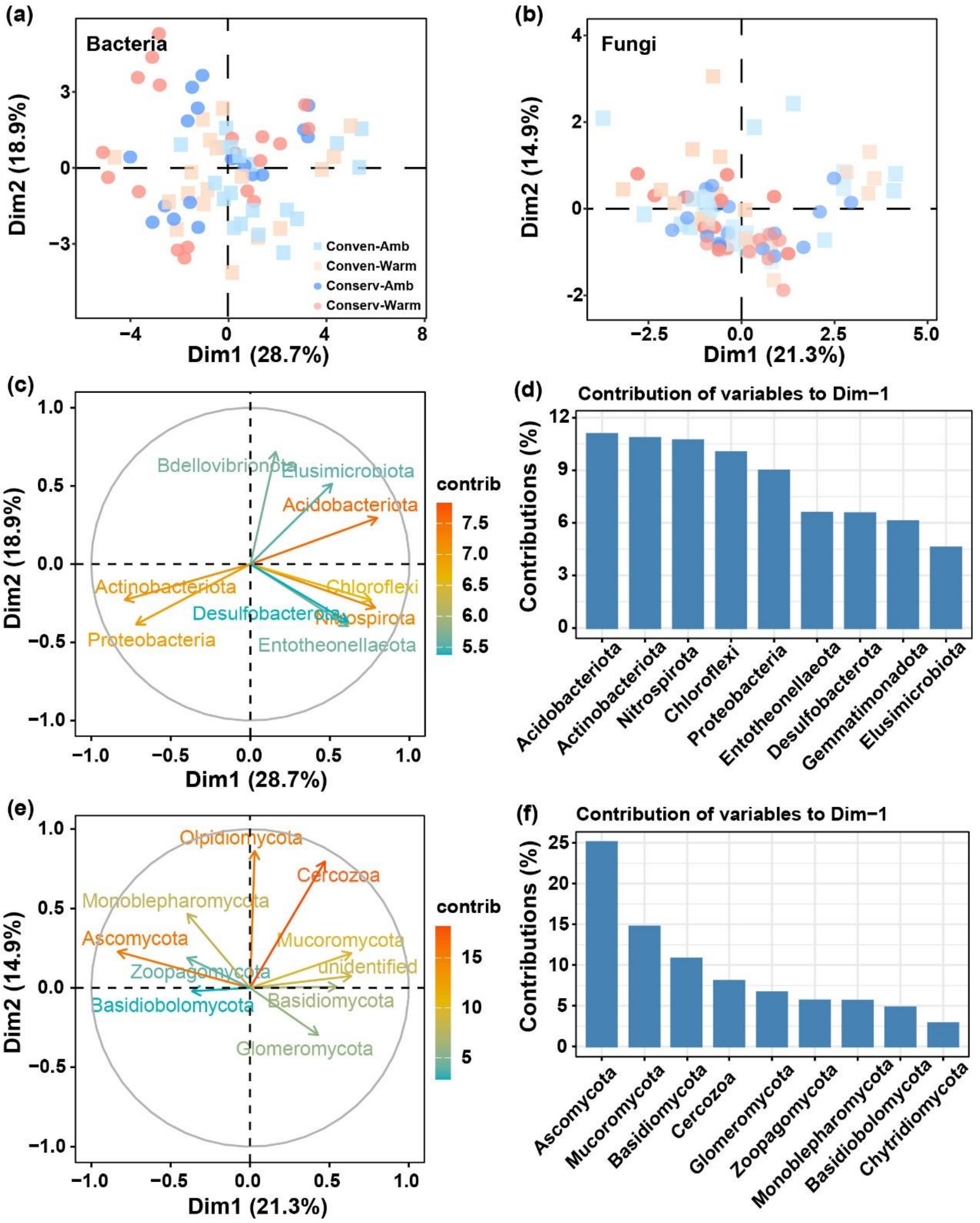

Supplementary Figure 6 Individual graphs of principal component analysis for bacterial and fungal community structure (a, b) and contributions of the main phylum for two dimensions of principal component analysis (c-f). Species distribution on the first two dimensions. The colors of the axis for the main species correspond to their contribution to the variation of the two dimensions. Conven-Amb: conventional agriculture without warming; Conven-Warm: conventional agriculture with warming; Conserv-Amb: conservation agriculture without warming; Conserv-Warm: conservation agriculture with warming.

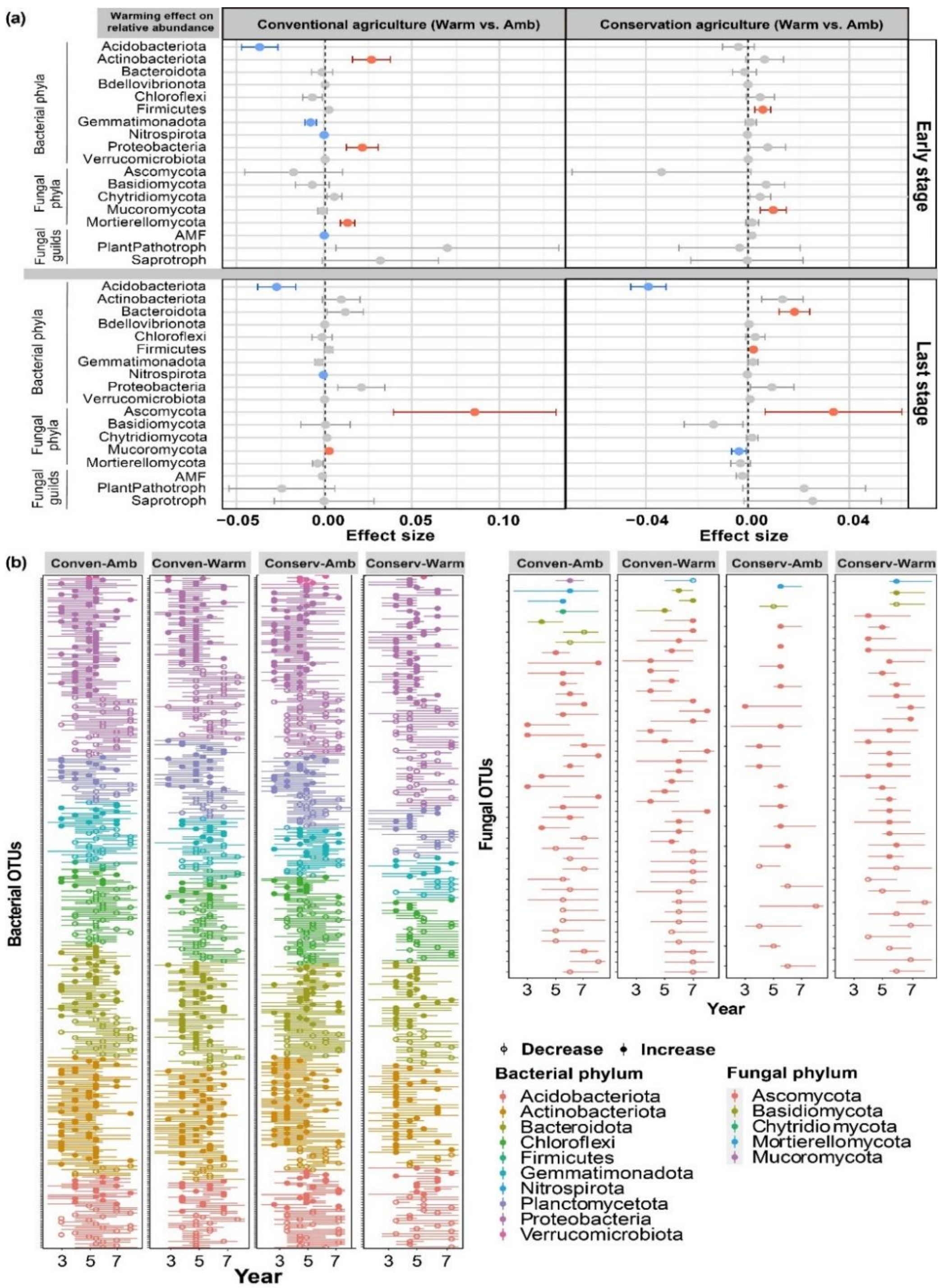

Supplementary Figure 7 Effect sizes of warming on the relative abundance of major microbial groups based on linear mixed-effects models (a). Data are presented as mean ± s.e.m. Statistical significance is based on Wald type II χ2 tests (n = 9 paired samples per management per stage); non-significant changes are denoted by grey dots.Two-sided statistical tests were used to evaluate the data. **Temporal change of individual OTU abundance shown by threshold indicator taxa analyses (TITAN)(b).** For each OTU, the taxonomic group is noted by color and filled symbols show declining abundance, and open symbols show increasing abundance. Statistical significance is based on Wald type II χ2 tests (n = 18 independent soil samples per treatment).

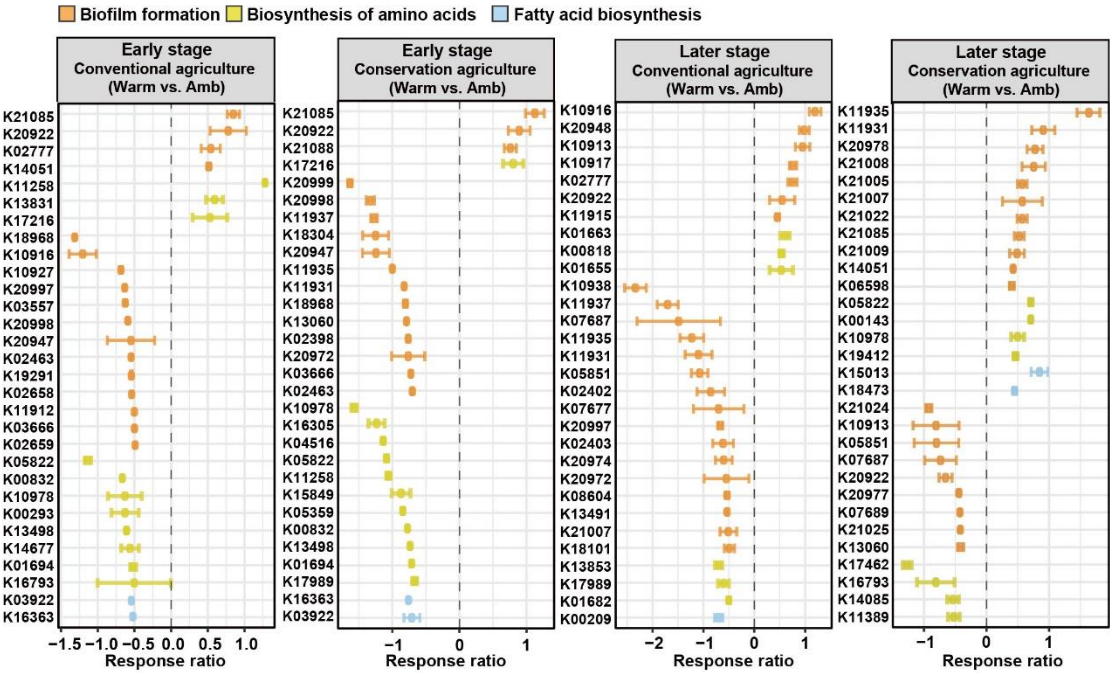

Supplementary Figure 8 Response ratios of soil microbial growth-related genes under two management systems based on the KEGG database. The top 30 genes that responded most strongly at different management practices and stages are shown (p < 0.05). Two-sided statistical tests were used to evaluate the data (n = 3 paired soil samples per management per stage). Conven-Amb: conventional agriculture without warming; Conven-Warm: conventional agriculture with warming; Conserv-Amb: conservation agriculture without warming; Conserv-Warm: conservation agriculture with warming.

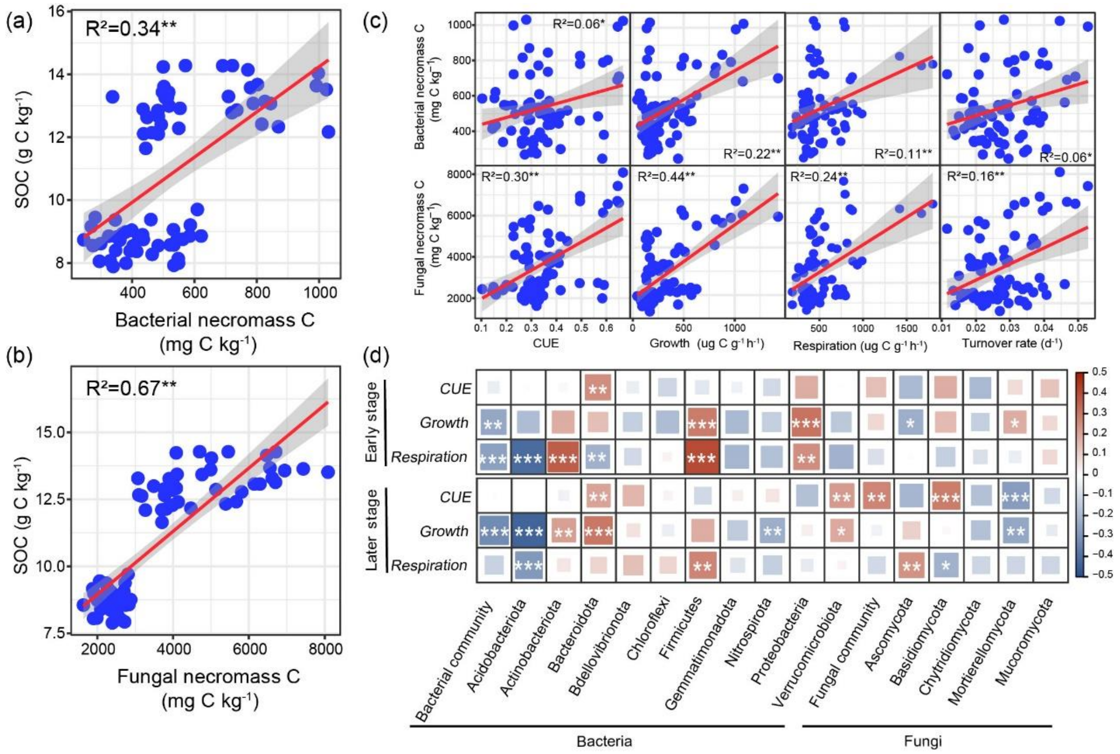

Supplementary Figure 9 Linear regression analysis showing relationships between microbial necromass, microbial physiology traits (CUE, growth, respiration and turnover rates), microbial community composition and SOC. The blue dots are individual measurements, the red fitted line is from linear regression. Only significant fitted lines are displayed on the graphs. The grey area is the 95% confidence interval of the fit. The color denotes the correlation coefficient determined by the linear mixed-effects model. Statistical significance is based on Wald type II χ^2 tests (two-sided) with n = 36 independent soil samples.

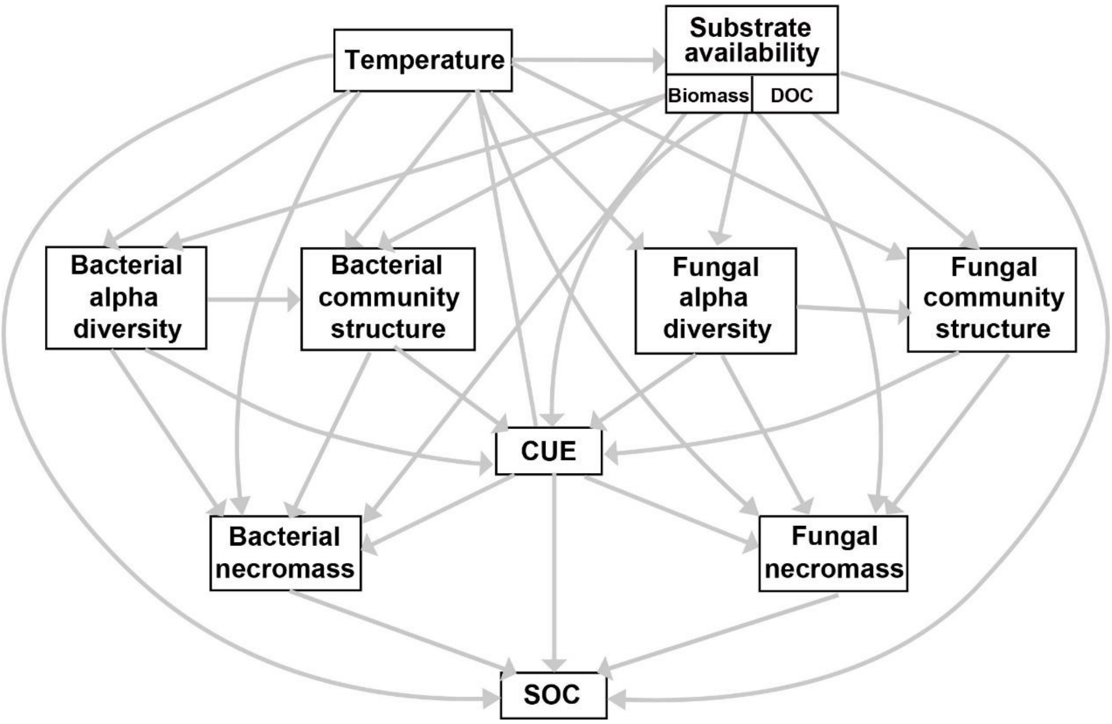

Supplementary Figure 10 Hypothesized conceptual models to evaluate microclimate, substrate availability, microbial diversity, physiological traits and SOC. We hypothesized that: 1) abiotic factors (microclimate and substrate availability) drive biotic factors (microbial CUE, diversity and necromass); 2) microbial alpha diversity and community structure drive the microbial CUE and necromass; 3) Microbial CUE drives necromass.

Supplementary Table 1 Results of repeated measures ANOVA analysis of the effects of management, warming, time and their interactions on soil SOC and microbial physiology

	SOC	Microbial growth	Respiration	C uptake	CUE
F					
Year (Y)	2.44	199	91.2	125	226
Management (M)	1849	216	36.5	152	98.6
Warming (W)	1.90	57.1	12.6	44.4	18.7
Y*M	4.06	26.5	5.59	19.8	7.51
Y*W	0.28	8.45	0.53	3.34	3.54
M*Y	6.99	16.8	0.11	5.92	0.01
Y*M*W	0.73	10.2	1.44	5.30	4.92
p					
Year (Y)	**0.042**	**<0.001**	**<0.001**	**<0.001**	**<0.001**
Management (M)	**<0.001**	**<0.001**	**<0.001**	**<0.001**	**<0.001**
Warming (W)	0.172	**<0.001**	**<0.001**	**<0.001**	**<0.001**
Y*M	**0.003**	**<0.001**	**<0.001**	**<0.001**	**<0.001**
Y*W	0.924	**<0.001**	0.752	**0.009**	**<0.001**
M*W	**0.010**	**<0.001**	0.745	**0.018**	0.933
Y*M*W	0.604	**<0.001**	0.221	**<0.001**	**<0.001**

Two-sided statistical tests were used to evaluate the data. Significant *p* values (< 0.05) are shown in bold.

Supplementary Table 2 Results (*p* values) of repeated measures ANOVAs on the effects of warming on SOC, soil temperature, moisture, aboveground biomass, root biomass, root exudation, DOC, total PLFAs, and the ratio of fungal and bacterial PLFAs under each management

Variables	Conven-Amb vs. Warm	Conserv-Amb vs. Warm
SOC	0.341	**0.026**
Soil temperature	**0.002**	**0.010**
Soil moisture	**< 0.001**	**< 0.001**
Aboveground biomass	0.115	**0.021**
Root biomass	**0.045**	**0.042**
Root exudation	**0.037**	**0.028**
DOC	0.452	**0.024**
Total PLFAs	0.820	**0.012**
Fungal PLFAs	0.378	**0.014**
Bacterial PLFAs	0.676	**0.008**
Fungal/bacterial PLFAs	0.317	**0.041**
Microbial CUE	0.465	**0.039**
Microbial growth	0.324	**< 0.001**
Microbial respiration	0.298	0.327
C uptake	0.139	**< 0.001**
Microbial necromass	0.778	**0.043**
Fungal necromass	0.956	**0.032**
Bacterial necromass	0.481	0.489
Fungal/bacterial necromass	0.821	0.153

Two-sided statistical tests were used to evaluate the data. Significant *p* values (< 0.05) are shown in bold.

Supplementary Table 3 Results of repeated measures ANOVA analysis of the effects of management, warming, time and their interactions on microbial necromass

	Bacterial necromass C	Fungal necromass C	Total necromass C	F/B necromass C
F				
Year (Y)	16.6	49.7	56.3	4.95
Management (M)	57.3	987	971	48.6
Warm (W)	2.14	40.1	39.2	1.21
Y*M	4.09	28.2	29.9	0.37
Y*W	1.47	8.91	9.15	0.94
M*Y	0.34	39.8	35.4	1.41
Y*M*W	0.93	6.97	6.41	1.89
p				
Year (Y)	**<0.001**	**<0.001**	**<0.001**	**0.001**
Management (M)	**<0.001**	**<0.001**	**<0.001**	**<0.001**
Warm (W)	0.150	**<0.001**	**<0.001**	0.2759
Y*M	**0.004**	**<0.001**	**<0.001**	0.868
Y*W	0.216	**<0.001**	**<0.001**	0.466
M*Y	0.562	**<0.001**	**<0.001**	0.240
Y*M*W	0.468	**<0.001**	**<0.001**	0.114

Two-sided statistical tests were used to evaluate the data. Significant *p* values (< 0.05) are shown in bold.

Supplementary Table 4 Permutational multivariate test for the slopes of microbial parameters response to warming under each management

Treatments	Pairwise Permanova (*p*)				
	Bacterial community	Fungal community	Bacterial necromass C	Fungal necromass C	Total necromass C
Conven-Amb vs. Warm	0.54	0.35	0.15	0.49	0.26
Conserv-Amb vs. Warm	0.92	**0.006****	0.45	**0.001****	**0.001****

Two-sided statistical tests were used to evaluate the data. Significant *p* values (< 0.05) are shown in bold.

Supplementary Table 5 Summary of permutational multivariate analysis of warming, management and year on microbial communities

	Adonis			
	Df	*F*	R^2	*p*
**Bacteria (16S)**				
Warming	1	4.48	0.021	**0.002**
Management	1	17.7	0.085	**0.001**
Time	5	19.5	0.47	**0.001**
Residuals			0.36	
**Fungi (ITS)**				
Warming	1	3.61	0.020	**0.005**
Management	1	14.5	0.080	**0.001**
Time	5	15.6	0.43	**0.001**
Residuals			0.42	

Two-sided statistical tests were used to evaluate the data. Significant *p* values (< 0.05) are shown in bold.

Supplementary Table 6 Significance tests of the effects of experimental warming on the microbial community structure over ten years across and within management system with three statistical approaches

	Adonis		Anosim		MRPP	
	F	*p*	*R*	*p*	*δ*	*p*
**All**						
Bacteria (16S)	4.48	**0.002**	0.026	**0.001**	0.38	**0.001**
Fungi (ITS)	3.61	**0.003**	0.019	**0.004**	0.60	**0.002**
**Traditional**						
Bacteria (16S)	4.86	**0.001**	0.056	**0.001**	0.34	**0.001**
Fungi (ITS)	3.48	**0.003**	0.038	**0.001**	0.60	**0.001**
**Conservation**						
Bacteria (16S)	3.88	**0.004**	0.008	**0.001**	0.37	**0.001**
Fungi (ITS)	2.62	**0.041**	0.003	**0.001**	0.55	**0.001**

All three tests are non-parametric multivariate analyses based on Bray–Curtis dissimilarities among samples. Two-sided statistical tests were used to evaluate the data. Significant *p* values (< 0.05) are shown in bold.

Supplementary Table 7 TDR values (*v*) of different phylogenetic groups based on taxonomic diversity under different treatments

Microbial Taxa	Treatments	*v*	*p*
**Bacteria**			
Acidobacteriota	Conven-Amb	-0.05	**0.002**
	Conven-Warm	-0.03	0.090
	Conserv-Amb	-0.04	0.060
	Convserv-Warm	-0.05	**0.050**
Actinobacteriota	Conven-Amb	-0.13	**<0.001**
	Conven-Warm	-0.09	**0.003**
	Conserv-Amb	-0.16	**<0.001**
	Convserv-Warm	-0.15	**<0.001**
Armatimonadota	Conven-Amb	-0.13	**0.002**
	Conven-Warm	-0.12	**0.019**
	Conserv-Amb	-0.11	0.060
	Convserv-Warm	-0.23	**<0.001**
Bacteroidota	Conven-Amb	-0.14	**<0.001**
	Conven-Warm	-0.12	**<0.001**
	Conserv-Amb	-0.09	**0.008**
	Convserv-Warm	-0.07	**0.040**
Chloroflexi	Conven-Amb	-0.13	**<0.001**
	Conven-Warm	-0.04	0.140
	Conserv-Amb	-0.20	**<0.001**
	Convserv-Warm	-0.18	**<0.001**
Firmicutes	Conven-Amb	-0.07	**0.019**
	Conven-Warm	-0.13	**0.020**
	Conserv-Amb	-0.05	0.220
	Convserv-Warm	0.02	0.740
Gemmatimonadota	Conven-Amb	-0.10	**<0.001**
	Conven-Warm	-0.09	**0.001**
	Conserv-Amb	-0.11	**<0.001**
	Convserv-Warm	-0.15	**<0.001**
Planctomycetota	Conven-Amb	-0.07	**<0.001**
	Conven-Warm	-0.06	**0.001**
	Conserv-Amb	-0.08	**<0.001**
	Convserv-Warm	-0.10	**<0.001**
Proteobacteria	Conven-Amb	-0.07	**0.003**
	Conven-Warm	-0.07	**0.005**
	Conserv-Amb	-0.09	**<0.001**
	Convserv-Warm	-0.07	**0.008**
Verrucomicrobiota	Conven-Amb	-0.04	0.530
	Conven-Warm	-0.10	0.120
	Conserv-Amb	-0.02	0.700

	Convserv-Warm	-0.02	0.660
**Fungi**			
Ascomycota	Conven-Amb	-0.32	**<0.001**
	Conven-Warm	-0.39	**<0.001**
	Conserv-Amb	-0.42	**<0.001**
	Convserv-Warm	-0.59	**<0.001**
Basidiomycota	Conven-Amb	-0.77	**<0.001**
	Conven-Warm	-0.54	**0.001**
	Conserv-Amb	-0.36	**0.050**
	Convserv-Warm	-0.89	**<0.001**
Chytridiomycota	Conven-Amb	-0.68	**0.017**
	Conven-Warm	-0.21	0.370
	Conserv-Amb	-0.71	**<0.001**
	Convserv-Warm	-0.51	**0.004**
Mortierellomycota	Conven-Amb	-0.06	0.85
	Conven-Warm	-0.10	0.13
	Conserv-Amb	0.15	0.11
	Convserv-Warm	-0.04	0.66
Mucoromycota	Conven-Amb	-0.45	**0.04**
	Conven-Warm	-0.20	0.42
	Conserv-Amb	-0.42	0.18
	Convserv-Warm	0.09	0.64

Two-sided statistical tests were used to evaluate the data. Significant *p* values (< 0.05) are shown in bold.

Supplementary Table 8 Permutational multivariate test for the TDR values (*v*) of different phylogenetic groups based on taxonomic diversity under different treatments

Phylogenetic groups	Convention Ambient vs.Warm	Conservation Ambient vs.Warm
Bacteria		
Acidobacteriota	0.181	0.767
Actinobacteriota	0.205	0.840
Armatimonadota	0.205	0.840
Bacteroidota	0.692	0.706
Chloroflexi	0.065	0.133
Firmicutes	**<0.001**	**<0.001**
Gemmatimonadota	**0.007**	0.112
Planctomycetota	0.779	**0.035**
Proteobacteria	0.940	0.250
Verrucomicrobiota	0.965	0.560
Fungi		
Ascomycota	0.202	**0.005**
Basidiomycota	0.189	**0.025**
Chytridiomycota	0.170	0.112
Mortierellomycota	**<0.001**	0.620
Mucoromycota	0.470	**0.001**

Two-sided statistical tests were used to evaluate the data. Significant p values (< 0.05) are shown in bold.

Supplementary Table 9 Correlations (*p* values) between microbial community and its phylogenetic lineages richness and physiological traits

	CUE	Growth	Respiration
Bacterial community	**0.004**	**<0.001**	**<0.001**
Acidobacteriota	0.858	**<0.001**	**<0.001**
Actinobacteriota	**0.018**	**<0.001**	**<0.001**
Bacteroidota	**0.001**	**<0.001**	0.109
Bdellovibrionota	**<0.001**	0.314	0.062
Chloroflexi	**<0.001**	**<0.001**	**0.011**
Firmicutes	**<0.001**	0.401	0.090
Gemmatimonadota	0.693	**<0.001**	**0.002**
Nitrospirota	0.321	**<0.001**	**0.002**
Proteobacteria	0.040	**<0.001**	**0.004**
Verrucomicrobiota	**<0.001**	0.023	0.100
Fungal community	**<0.001**	**<0.001**	**0.016**
Ascomycota	**<0.001**	**<0.001**	**0.012**
Basidiomycota	**<0.001**	**0.028**	0.358
Chytridiomycota	0.185	0.066	0.147
Mortierellomycota	0.087	0.136	0.221
Mucoromycota	0.054	0.072	0.221

Two-sided statistical tests were used to evaluate the data. Significant *p* values (< 0.05) are shown in bold.

Supplementary Table 10 Correlations (*p* values) between carbon metabolism-associated functions of soil microorganisms and physiological traits

	CUE	Growth	Respiration	Necromass
Biofilm formation	**0.035**	**0.006**	**0.036**	**0.048**
Fatty acid biosynthesis	0.060	**<0.001**	**0.002**	**0.007**
Biosynthesis of aminoacids	0.768	0.218	0.039	0.885
Arginine biosynthesis	0.514	**0.046**	**0.016**	0.686
Glucosinolate biosynthesis	**0.011**	**<0.001**	**<0.001**	**0.008**
Valine.leucine and isoleucine biosynthesis	**0.005**	**<0.001**	**<0.001**	**0.009**
Phenylalanine.tyrosine and tryptophan biosynthesis	**0.045**	**<0.001**	**<0.001**	**0.034**
Lysine biosynthesis	**0.042**	**<0.001**	**<0.001**	**0.016**
Pentose and glucuronate interconversions	**0.047**	**0.001**	**<0.001**	**0.014**
Pyruvate metabolism	**0.035**	**0.007**	**0.046**	**0.029**
Pentose phosphate pathway	**0.019**	**<0.001**	**<0.001**	**0.012**
starch	**0.007**	**<0.001**	**0.000**	**0.017**
pectin	0.465	0.117	**0.023**	0.188
cellulose	0.360	0.189	0.188	0.340
chitin	**0.002**	**<0.001**	**<0.001**	**0.003**
lignin	0.104	**0.001**	**<0.001**	**0.015**

Two-sided statistical tests were used to evaluate the data. Significant *p* values (< 0.05) are shown in bold.
