# Supplementary material for: Microbially mediated mechanisms underlie soil carbon accrual by conservation agriculture under decade-long warming
Source: Nat Commun. 2024 Jan 8;15:377. doi: 10.1038/s41467-023-44647-4 (PMC10774409; doi:10.1038/s41467-023-44647-4)
Supplement: Supplementary file 3 — Reporting Summary [file 41467_2023_44647_MOESM3_ESM.pdf]

nature portfolio

Corresponding author(s): Jizhong Zhou

Last updated by author(s): Jun 1, 2023

Reporting Summary

Nature Portfolio wishes to improve the reproducibility of the work that we publish. This form provides structure for consistency and transparency in reporting. For further information on Nature Portfolio policies, see our Editorial Policies and the Editorial Policy Checklist.

Statistics

For all statistical analyses, confirm that the following items are present in the figure legend, table legend, main text, or Methods section.

n/a	Confirmed	
☐	☒	The exact sample size (*n*) for each experimental group/condition, given as a discrete number and unit of measurement
☐	☒	A statement on whether measurements were taken from distinct samples or whether the same sample was measured repeatedly
☐	☒	The statistical test(s) used AND whether they are one- or two-sided<br>*Only common tests should be described solely by name; describe more complex techniques in the Methods section.*
☐	☒	A description of all covariates tested
☐	☒	A description of any assumptions or corrections, such as tests of normality and adjustment for multiple comparisons
☐	☒	A full description of the statistical parameters including central tendency (e.g. means) or other basic estimates (e.g. regression coefficient) AND variation (e.g. standard deviation) or associated estimates of uncertainty (e.g. confidence intervals)
☐	☒	For null hypothesis testing, the test statistic (e.g. *F*, *t*, *r*) with confidence intervals, effect sizes, degrees of freedom and *P* value noted<br>*Give P values as exact values whenever suitable.*
☒	☐	For Bayesian analysis, information on the choice of priors and Markov chain Monte Carlo settings
☐	☒	For hierarchical and complex designs, identification of the appropriate level for tests and full reporting of outcomes
☐	☒	Estimates of effect sizes (e.g. Cohen's *d*, Pearson's *r*), indicating how they were calculated

Our web collection on statistics for biologists contains articles on many of the points above.

Software and code

Policy information about availability of computer code

Data collection	No software used for data collection.
Data analysis	UNOISE3,Trimmomatic (V 0.33), FALSH (V1.2.11), UPARSE, RDP Classifier, and MEGAHIT (V1.1.2) were used to process the sequencing data; R (V 3.2.1) and AMOS 23.0 were used for statistical analyses.<br>The R packages used statistical analyses include smatr, effsize, vegan, lme4, and car.<br>The analysis code that supports the findings of this study is available at GitHub https://github.com/bio-carbon/code.

For manuscripts utilizing custom algorithms or software that are central to the research but not yet described in published literature, software must be made available to editors and reviewers. We strongly encourage code deposition in a community repository (e.g. GitHub). See the Nature Portfolio guidelines for submitting code & software for further information.

Data

Policy information about availability of data

All manuscripts must include a data availability statement. This statement should provide the following information, where applicable:

- Accession codes, unique identifiers, or web links for publicly available datasets
- A description of any restrictions on data availability
- For clinical datasets or third party data, please ensure that the statement adheres to our policy

The authors declare that the data supporting the findings of this study are availability with the article and its supplementary Information files, and from the

corresponding authors on request. The DNA sequences of the 16S rRNA gene and ITS amplicons in this study have been deposited in the National Center for Biotechnology Information (NCBI) under project accession numbers PRJNA903096 and PRJNA903090. Raw shotgun metagenomic sequences in this study have been deposited in the National Center for Biotechnology Information (NCBI) under project accession PRJNA1007786. Silva database is available at https://www.arb-silva.de/. UNITE database is available at https://unite.ut.ee/. Source data are provided in this paper.

Research involving human participants, their data, or biological material

Policy information about studies with human participants or human data. See also policy information about sex, gender (identity/presentation), and sexual orientation and race, ethnicity and racism.

Reporting on sex and gender	N/A
Reporting on race, ethnicity, or other socially relevant groupings	N/A
Population characteristics	N/A
Recruitment	N/A
Ethics oversight	N/A

Note that full information on the approval of the study protocol must also be provided in the manuscript.

Field-specific reporting

Please select the one below that is the best fit for your research. If you are not sure, read the appropriate sections before making your selection.

☐ Life sciences ☐ Behavioural & social sciences ☒ Ecological, evolutionary & environmental sciences

For a reference copy of the document with all sections, see nature.com/documents/nr-reporting-summary-flat.pdf

Ecological, evolutionary & environmental sciences study design

All studies must disclose on these points even when the disclosure is negative.

Study description	This study investigated temporal effects of warming and management interactions on SOC persistence, as well as identified the microbial attributes underlying the response. The study is a long-term manipulated field experiment with four treatments: conservation agriculture with and without warming (Conserv-Amb, Conserv-Warm) and conventional agriculture with and without warming (Conven-Amb, Conven-Warm). Two levels of warming (ambient and +2°C) were imposed on both conventional agriculture and conservation agriculture since 2010.
Research sample	The differences of warmed and control treatments under two managements represent the effects of long-term experimental warming and management interactions on ecosystem functions and soil microbial attributes.
Sampling strategy	Soil samples were taken using a soil corer (5 cm inner diameter) in 2010, 2012, 2014, 2016, 2018 and 2020. Five soil cores in each plot and composited to have enough samples for soil chemistry, microbiology and molecular biology analyses. Before microbial and chemical analyses, the composited soil samples were passed through a 2 mm sieve to remove visible roots and gravel.
Data collection	All sample collection from the experiment site was performed by authors JT and RXH. Soil chemistry, and measurements of soil moisture, temperature, root biomass and rhizodeposiiton were performed by the JT following standard protocols. Soil DNA extraction and PCR were performed by JT. The amplicon sequencing and Metagenomic sequencing were performed on an Illumina Nova6000 and Illumina NovaSeq at Majorbio Bio-Pharm Technology Co., Ltd. (Shanghai, China).
Timing and spatial scale	SOC, TN, and DOC were measured after sample collection in 2010, 2012, 2014, 2014, 2018 and 2020. Microbial CUE and diversity were measured in 2021 in all soils (sampled in 2010, 2012, 2014, 2016, 2018 and 2020) that had been stored at -80°C . The metagenomic sequencing were only used samples from 2010 and 2020. The samples for root rhizodeposition were conducted from October 2019 to May 2020 during winter wheat growth season.
Data exclusions	No data were excluded.
Reproducibility	16S rRNA gene and ITS amplicons were sequenced on an Illumina Nova6000 platform according to the standard protocols by Majorbio Bio-Pharm Technology Co. Ltd. (Shanghai, China).<br>Metagenomic sequencing was performed on Illumina NovaSeq at Majorbio Bio-Pharm Technology Co., Ltd. (Shanghai, China).<br>The microcosm experiments data are generated from four replicates and multiple time points.
Randomization	Four treatments were laid out in a randomized complete block design with four replicates: conservation agriculture with and without warming (Conserv-Amb, Conserv-Warm) and conventional agriculture with and without warming (Conven-Amb, Conven-Warm).

Blinding	All samples taken were labeled with a single number to track samples during lab processing, but included no information as to the treatment from which it originated.

Did the study involve field work? ☒ Yes ☐ No

Field work, collection and transport

Field conditions	A long-term field experiment with a double-cropped winter wheat (Triticum aestivum L.)-summer maize (Zea mays L.) system was located at the Yucheng Comprehensive Experiment Station in North China (36°51′N, 116°34′E), which belong to the Chinese Academy of Science (CAS). The region has a temperate semi-arid climate with an annual mean temperature of 13.1°C, and annual mean precipitation of 561mm. The soil has a silt loam texture with 12% sand, 66% silt, 22% clay, and a mean pH of 7.1. The soil type is Calcaric Fluvisol according to the FAO-UNESCO system.
Location	The Yucheng Comprehensive Experiment Station in North China (36°51′N, 116°34′E).
Access & import/export	Project and class site use requests were completed for our study.
Disturbance	No disturbance was caused by this study.

Reporting for specific materials, systems and methods

We require information from authors about some types of materials, experimental systems and methods used in many studies. Here, indicate whether each material, system or method listed is relevant to your study. If you are not sure if a list item applies to your research, read the appropriate section before selecting a response.

Materials & experimental systems

n/a	Involved in the study	
☒	☐	Antibodies
☒	☐	Eukaryotic cell lines
☒	☐	Palaeontology and archaeology
☒	☐	Animals and other organisms
☒	☐	Clinical data
☒	☐	Dual use research of concern
☒	☐	Plants

Methods

n/a	Involved in the study	
☒	☐	ChIP-seq
☒	☐	Flow cytometry
☒	☐	MRI-based neuroimaging
